# The effect of Covid-19 on the willingness to use video consultations among orthopedic and trauma outpatients: a multi-center survey in 1400 outpatients

**DOI:** 10.1007/s00068-021-01774-1

**Published:** 2021-08-24

**Authors:** Julian Scherer, David A. Back, Friedrich Thienemann, Ernest Kaufmann, Valentin Neuhaus, Christian Willy, Pierre Hepp, Hans-Christoph Pape, Georg Osterhoff

**Affiliations:** 1 Department of Traumatology, University Hospital of Zurich, Raemistrasse 100, 8091 Zurich, Switzerland; 2 Bundeswehr Hospital Berlin, Clinic of Traumatology and Orthopedics, Berlin, Germany; 3 Department of Medicine, University Hospital of Zurich, Raemistrasse 100, 8091 Zurich, Switzerland; 4 Department of Urology, University Hospital of Zurich, Raemistrasse 100, 8091 Zurich, Switzerland; 5 Department of Orthopaedics, Trauma and Plastic Surgery, University Hospital of Leipzig, Liebigstr. 20, 04103 Leipzig, Germany

**Keywords:** Covid-19, Survey, Telehealth, Video consultation, Orthopedic trauma, Trauma, attitude

## Abstract

**Introduction:**

Due to the Covid-19 pandemic, making more use of remote medical solutions has been advertised. The purpose of this study was to assess the willingness of orthopedic trauma patients to conduct an online video consultation (OVC) during the coronavirus pandemic compared to before.

**Methods:**

A survey amongst orthopedic and trauma outpatients from three European trauma centers was conducted via paper-based questionnaires, composed of participants’ demographics and five open and closed questions between June and November 2019 and between April and July 2020 during Covid-19. The main outcome was the difference between the cohorts regarding the willingness to use an OVC, reasons for and against usage as well as advantages and disadvantages. Sub-analysis was performed for gender, participants’ occupation and three age groups (≤ 30 years; 31–55 years; > 55 years).

**Results:**

1400 participants (780 preCovid-19 and 620 Covid-19) were included. There was no difference in willingness to conduct an OVC between the cohorts (57.6% versus 63.9%; *p* = 0.053). The highest disposition towards an OVC in both cohorts was seen in patients below 30 years of age, followed by 31–55 years and over 55 years. Women were significantly more likely in the Covid-19-group than in the preCovid-19-group to conduct an OVC (*p* = 0.032). Use of the OVC for “personal questions to the physician” was more often stated in the Covid-19-cohort (*p* = 0.007). “No danger of an infection” (*p* = 0.001) and “availability from anywhere” (*p* = 0.032) as advantages of an OVC were more often stated in the Covid-19-cohort. “No direct contact with the doctor” (*p* = 0.001) and “relationship to the doctor could change” (*p* = 0.024) as disadvantages of the OVC were less often stated in the Covid-19-cohort.

**Conclusion:**

The majority of the assessed outpatients would use an OVC. Fear of infections have increased, and direct physical contact is less important since the Covid-19 pandemic, but have not increased the disposition for an OVC significantly.

## Introduction

Since the outbreak of the coronavirus disease 2019 (Covid-19), several health authorities have proposed a social gap, including reduction of interpersonal contact, to minimize the spread of the disease [1, 2]. A potential solution to this is online communication. Especially, during the pandemic, the rapid use of telemedicine amongst all medical disciplines has shown its potential as a promising tool to deliver care remotely [3, 4]. Whilst online consultations have shown their potential long before the pandemic, they have not yet been implemented into daily clinical life. Due to the Covid-19 crisis, health systems implemented this service quickly to satisfy as many patients possible while obeying to social-distancing rules [5]. Despite the general opinion, that surgical disciplines are exclusively hands-on and, therefore, would not suite for telemedical therapy or follow-up, several studies have shown its feasibility for remotely delivered care pre- and post-surgically [6–8]. While telehealth can be considered as a broad field of remote service, especially online video consultations (OVC) can replace physical visits for initial evaluation, pre-surgical prep as well as post-surgical follow-up and has been shown to satisfy patients as well as practitioners adequately [6, 9–11]. In orthopedic trauma, video consultations can be utilized for initial assessment of minor injuries, post-surgical rehabilitation and follow-up, as well as pre-surgical assessment [4, 12]. Furthermore, it has been shown, that especially in orthopedic trauma, there is no difference in terms of satisfaction between in person and online consultation amongst patients and physicians and that certain video-tools and applications can be equally effective as a face-to-face physical examination during the Covid-19 pandemic [13, 14]. It has been shown, that during the corona crisis, telemedicine can be implemented into clinical every-day-work-life rapidly and to a high percentage of satisfaction amongst orthopedic trauma patients and surgeons [15]. Seeing a relatively high welcomeness towards online and especially video consultations amongst patients and physicians before the outbreak of the coronavirus, it is not known if, and to what extent, the pandemic had an influence on the attitude towards telehealth [16–18]. Thus, the aim of this study was to assess the willingness of orthopedic and trauma patients to perform a hypothetical remote video consultation in times of the Covid-19 pandemic compared to before and to identify factors which might have changed this attitude due to the pandemic.

## Methods and materials

### Patients and survey design

A standardized questionnaire was handed out to patients in the outpatient clinics of the authors’ trauma and orthopedic departments in Switzerland (University Hospital of Zurich) and Germany (Bundeswehr Hospital Berlin, University Hospital of Leipzig). None of these centers were offering remote video consultations regularly at the time of the survey. The survey was first conducted between June 2019 to November 2019 and was published consecutively [4]. The exact same questionnaire was handed out again to outpatients in the respective departments during April and July 2020 (Covid-19-pandemic), without mentioning the pandemic or the comparison character of the second part of the study to minimize the possibility of bias. The only inclusion criterion was being currently treated by an orthopedic or trauma surgeon at one of those three institutions.

### Ethics approval statement

Participation was voluntary and anonymity was granted. All participants received a written patient information explaining the aim of the study and processing of their data. No identifying data except for age, gender and occupation were collected. Hence, data can be assumed to be anonymous and the European data protection regulations do not apply. In addition, the local ethics committees of all participating centers have declared general waivers for surveys with anonymous data. By answering the questionnaire, participants gave consent to the use of the data that they had provided.

### Study questionnaire

The questionnaire first explained the general process and the technical requirements for a successful OVC. In the following, the participants’ baseline characteristics including age, sex and professional status (part-time, “9-to-5”, self-employed, shift-work, others and “retired”) were obtained. They were then asked, if they would use a video consultation in general and if they have the equipment for conducting a video consultation (yes, no, uncertain). In further three questions, patients were asked for what special reason they would use it and what advantages and disadvantages they see in a video consultation by choosing from a number of answers provided, including optional free text comments.

### Statistical analysis

Further statistical analysis was done by the use of SPSS for Mac 25.0 (SPSS, Chicago, Illinois, USA). Data are presented as frequencies (*n*) and means with the standard deviation (SD). To assess differences between groups, a chi-squared test was used for categorical data. A subgroup analysis was performed for age (< 30 years, 30–55 years, > 55 years, arbitrary selection of thresholds), gender and profession. The thresholds for the assignment of participants to the specific age groups were chosen based on a previous study for reasons of better comparability. These thresholds were originally chosen in view of expected differences in mobility and the ability to use digital (health) applications. The level of statistical significance was set at *p* < 0.05.

### Patient and public involvement

In this study, patients or the public were not involved in the design, or conduct, or reporting, or dissemination plans of our research.

## Results

### Demographics

In total, we included 780 participants (preCovid-19-cohort) (302 female, 38.7%, 478 males, 61.3%) before Covid-19 (June–November 2019) with a mean age of 43.8 years (SD 17.1, range from 14 to 94 years). During the Covid-19 pandemic (April–June 2020), we included 620 participants (Covid-19-cohort) (219 female, 35.3%, 301 males, 64.7%) with a mean age of 43.7 years (SD 17.5, range from 16 to 89 years). There was no significant difference between the two cohorts (Table 1).

**Table 1:** Demographics of assessed participants stratified by cohort (PreCovid-19 vs. Covid-19), age group and gender

Group	PreCovid-19		Covid-19		Total	*p* value
	Female	Male	Female	Male		
< 30 years	70	150	59	119	398 (28.4%)	0.778
31–55 years	125	231	88	190	634 (45.3%)	0.360
** > **55 years	107	97	72	92	368 (26.3%)	0.103
Total	302 (38.7%)	478 (61.3%)	219 (35.3%)	401 (64.7%)		

In both cohorts, most participants had a “9-to-5” job (32.9 vs. 35.5%), followed by “others” (22.8 vs. 20.6%), “retired” (16.9 vs. 19.9%), “shift-work” (16.3 vs. 12.7%), “part-time” (6.5 vs. 6.8%) and “self-employed” (4.5 vs. 5.4%) with no significant differences between the cohorts (*p* = 0.340).

#### Question 1: Could you generally imagine using a video consultation?

**Fig. 1:**
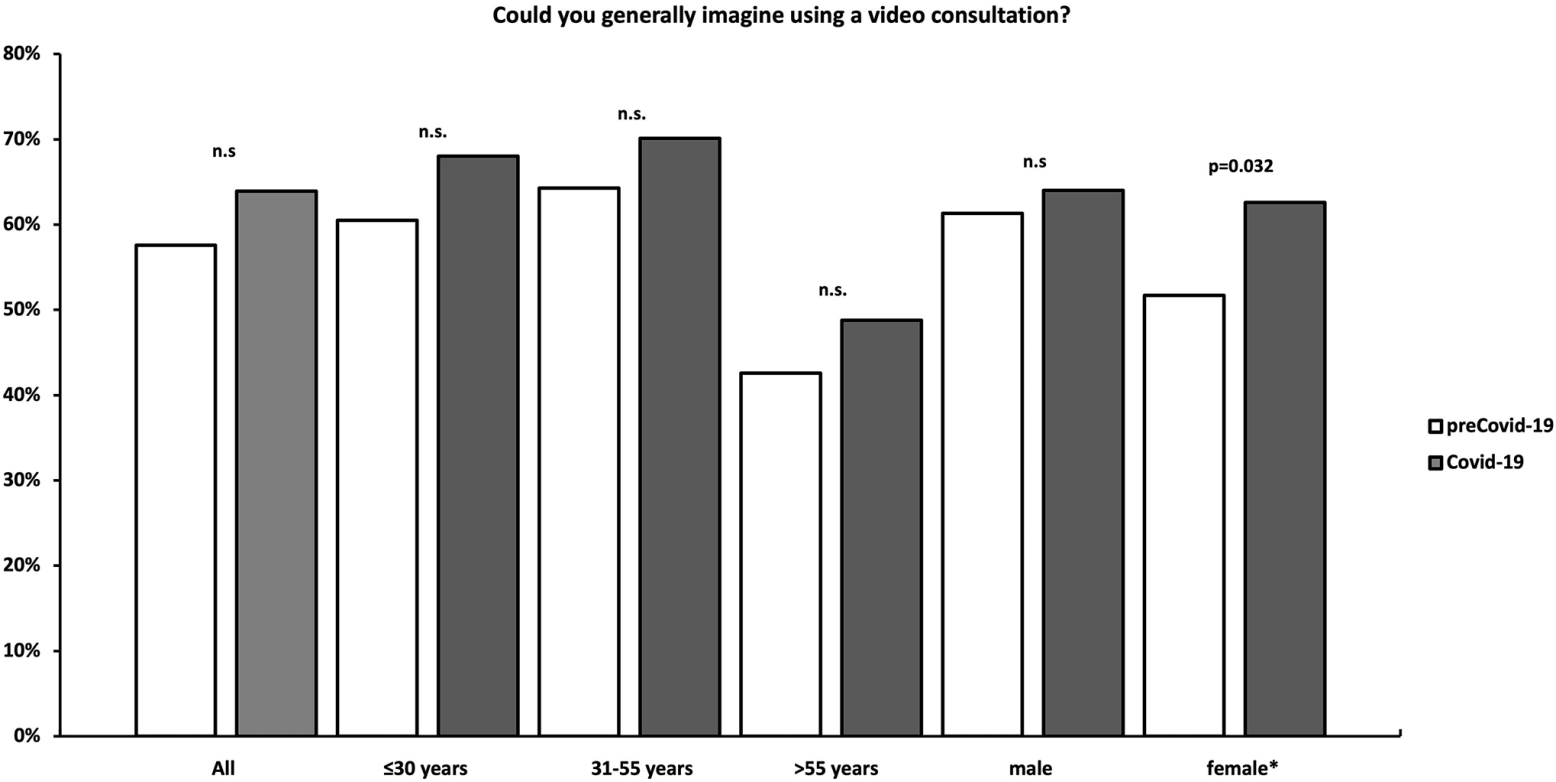
Willingness to perform a video consultation stratified by age and gender. *n.s.* not significant

**Fig. 2:**
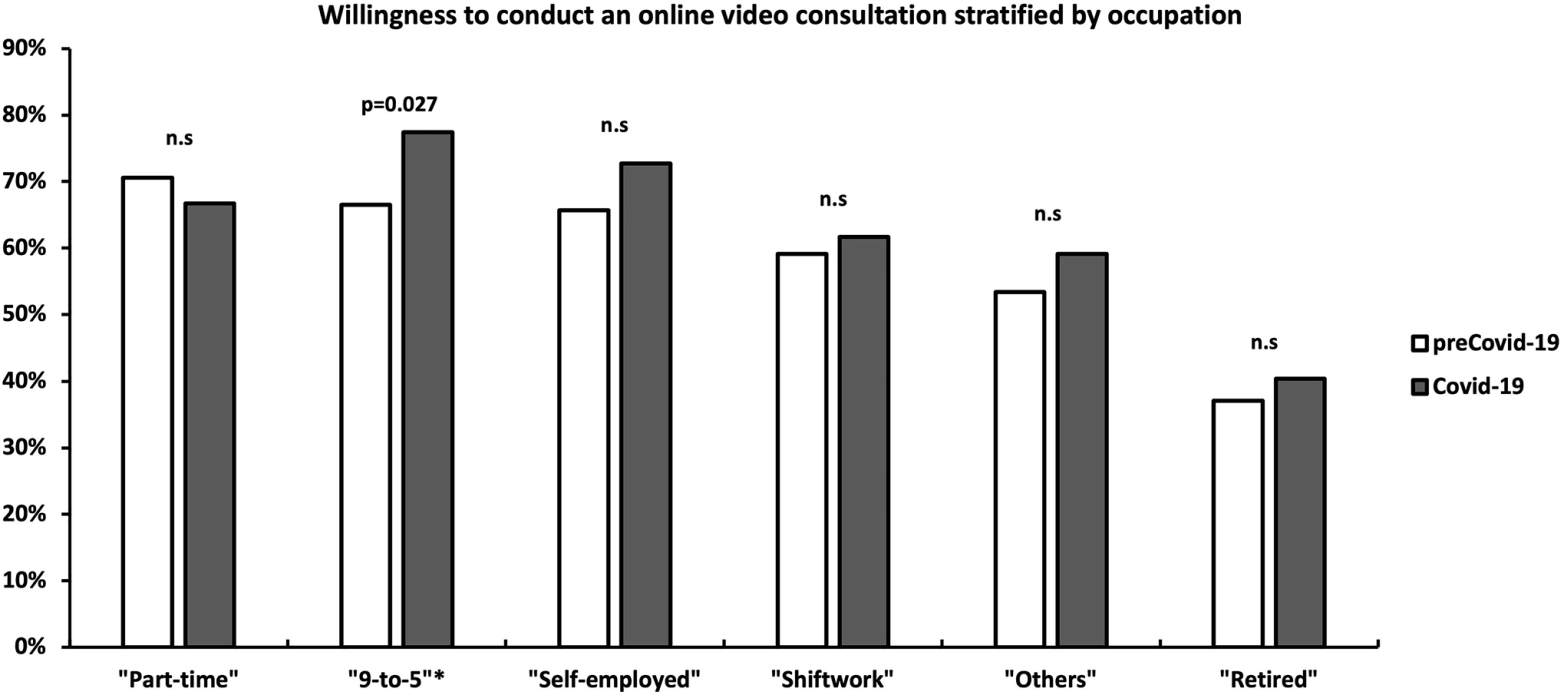
Willingness to perform a video consultation stratified by occupation. *n.s*. not significant

Participants in the Covid-19 cohort were not significantly more willing to use a video consultation (*n* = 396, 63.9%) than participants in the preCovid-19 cohort (*n* = 449, 57.6%) (*p* = 0.053). In total, 21.3% (*n* = 166) (preCovid-19) and 18.7% (*n* = 116) (Covid-19) were not eager to use a remote consultation and 21.2% (*n* = 165) (preCovid-19) and 17.4% (*n* = 108) in (Covid-19) were unsure (*p* = 0.053). There was no significant difference between the assessed cities (Zurich, Berlin, Leipzig) the survey was conducted in (*p* = 0.889). In the preCovid-19-cohort, males were more willing to conduct a remote consultation than females (*p* = 0.027), whereas in the Covid-19-cohort, there was no significant difference between male and female participants found (*p* = 0.274). Women were significantly more likely in the Covid-19-group (62.6%) than in the preCovid-19-group (51.7%) to conduct a video consultation (*p* = 0.032). In both cohorts, the highest willingness to perform a remote consultation amongst the assessed age groups was found in age group 31–55 years) (64.3 vs. 70.1%, *p* = 0.125), followed by age group ≤ 30 years (60.5 vs. 68%, *p* = 0.078) and age group > 55 years (42.6 vs. 48.8%, *p* = 0.450) (Fig. 1). The willingness of conducting an OVC stratified by the participants’ occupation is shown in Fig. 2.

#### Question 2: Do you own a device suitable for a video consultation (e.g. smartphone, laptop with camera and microphone)?

In the preCovid-19-group, 86.2% of the participants and 88.7% of the participants in the Covid-19-group stated that they had a smartphone and/or another device (laptop, tablet) with which a video consultation would be possible (camera, microphone, internet) (*p* = 0.137). In the group of patients with an age below 30 years, the pre-Covid-19 cohort had an incidence of smartphone and/or other compatible devices of 93.6%, whereas the Covid-19 cohort had an incidence of 99.4% (*p* = 0.024). In the group with patients aged 31–55 years, 94.1% had the required technology in the pre-Covid-19 cohort, compared to 93.9% in the Covid-19 cohort (*p* = 0.270). Participants older than 55 years of age (age group 3) showed a decreased usage of digital technology, suitable for conducting a remote consultation (64.2 vs. 68.3%) (*p* = 0.306).

#### Question 3: For what specific situation could you imagine using the video consultation?

**Fig. 3:**
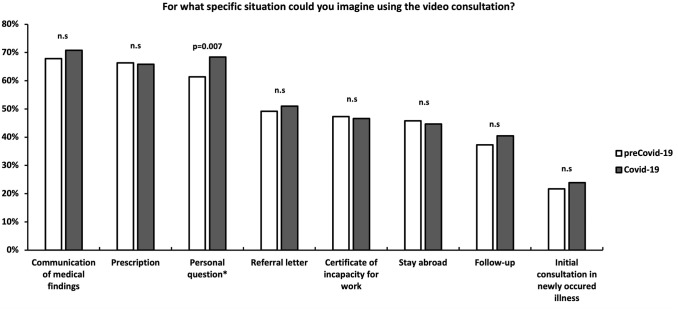
Specific situations an online video consultation would be used for. *n.s.* not significant

Participants from the Covid-19-cohort stated that they would use the proposed OVC for a “personal question to the treating physician” significantly more often than their counterparts from the preCovid-19-cohort (61.4 vs. 68.4%, *p* = 0.007) (Fig. 3).

#### Question 4: Which of the mentioned advantages do you see in the video consultation?

**Fig. 4:**
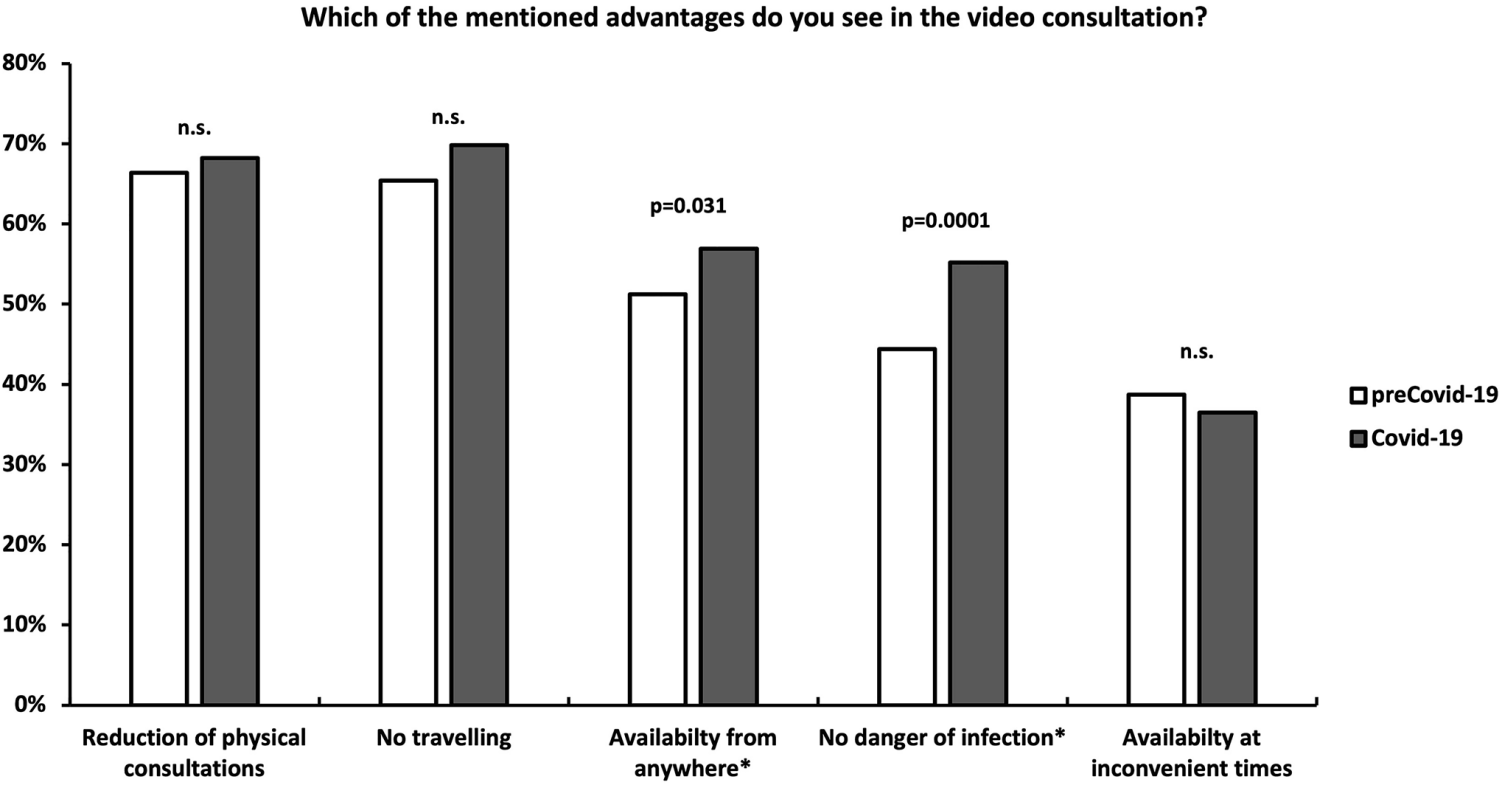
Advantages of an online video consultation. *n.s.* not significant

“Availability from anywhere” (51.2 vs. 56.9%, *p* = 0.031) and “no danger of infection” (44.4 vs. 55.2%, *p* = 0.001) were factors in favor of the video consultation significantly more often stated by the outpatients from the Covid-19-cohort (Fig. 4).

#### Question 5: Which of the mentioned disadvantages do you see in the video consultation?

**Fig. 5:**
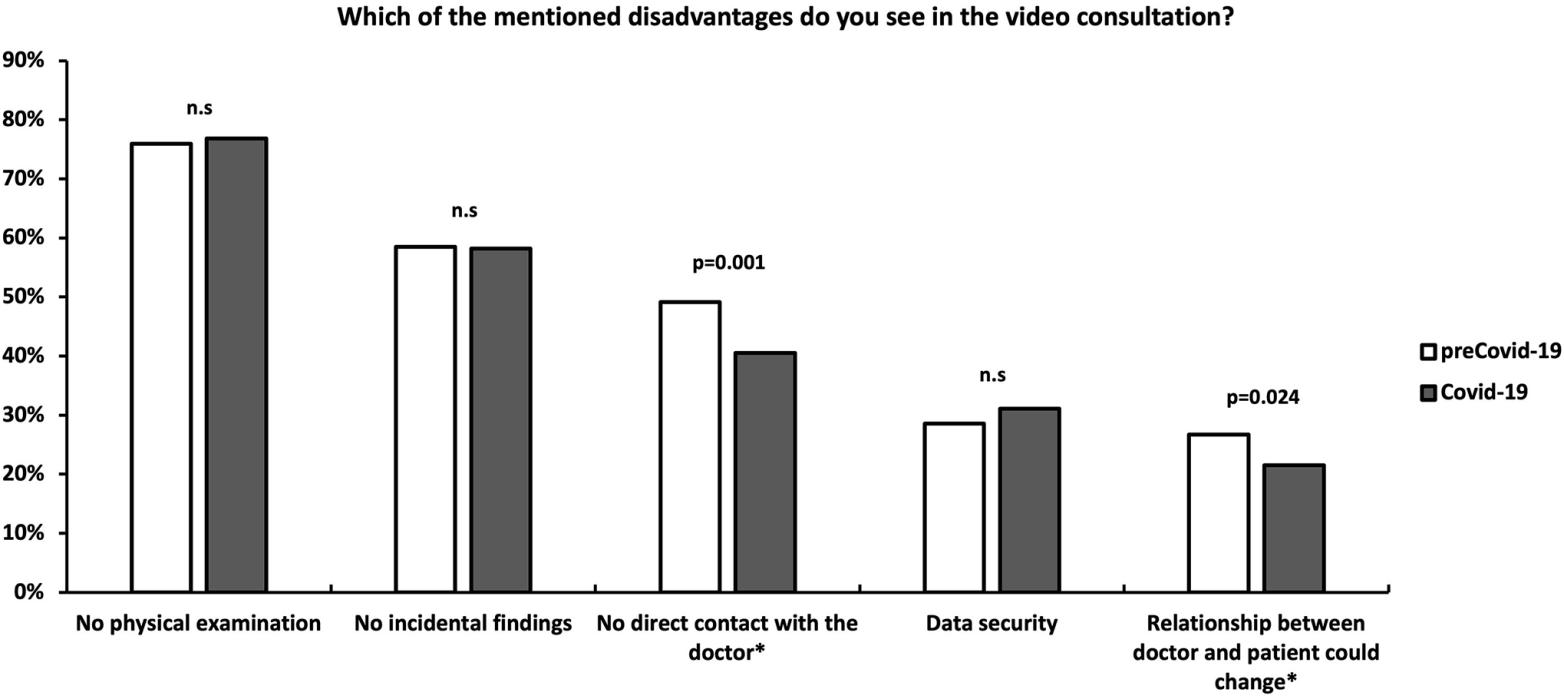
Disadvantages of an online video consultation. *n.s.* not significant

“No direct contact with the doctor” (49.1 vs. 40.5%, *p* = 0.001) and “relationship between doctor and patient could change” (26.7 vs. 21.5%, *p* = 0.024) as potential disadvantages of the OVC were significantly less often stated by outpatients from the Covid-19-cohort (Fig. 5).

## Discussion

The aim of the present study was to assess the general willingness to conduct a hypothetical remote video consultation amongst orthopedic and trauma outpatients during the Covid-19 pandemic and compare these findings to the “normal” times before the coronavirus crisis to identify the effect of the novel Sars-CoV-2 on patients’ disposition towards telemedicine. Furthermore, we tried to identify new aspects in favor and against conducting a video consultation out of the patients’ perspective, which may have evolved during this unusual time.

To exclude bias, we used exactly the same questionnaire as in a previous study, without mentioning the pandemic or the comparing character of the study [4]. Afterwards, the two cohorts, preCovid-19 and Covid-19, were compared.

The two respected cohorts had no significant differences in regards of their demographic baseline characteristics.

In our study population, there was no significant difference in terms of willingness to conduct a hypothetical OVC between the Covid-19-cohort and the preCovid-19-cohort amongst all assessed age groups. Since lockdown has been established in many countries around the world due to the pandemic, several studies on patients’ satisfaction with online consultations, mainly but not exclusively performed with general practitioners, have been carried out and suggested good satisfaction levels amongst patients and physicians [19–21]. Data show that the number of conducted OVCs during lockdown has risen dramatically in European Countries like Germany, Spain, UK and France and, therefore, reflects a higher general willingness of patients to conduct remote consultations since the corona crisis [5, 22]. However, to our knowledge, no comparative study, which examines the effect of the Covid-19 pandemic on the willingness of “naive” patients, especially in the field of trauma and orthopedic surgery, exists to date. Interestingly, the youngest age group in our study population did not show the highest inclination towards conducting a remote video consultation, which we conclude as a result of a higher awareness of the potential downsides and data security concerns while transducing digital data. Interestingly, we found a higher willingness towards conducting a remote video consultation in the Covid-19-cohort amongst women. This may be a result of different coping strategies in face of the threat of a Covid-19-infection, which have been found to be gender-dependent with women being more effected than men [23, 24].

In the Covid-19 cohort, we found a significantly higher disposition to conduct remote consultations amongst patients occupied in a “9-to-5” work environment. We interpret this finding as a result of home-office environment and a rising acceptance of video-calls in working-life.

In terms of smartphone-usage, we found a rising number of users amongst all ages, especially within the group of under 30 years of age, which can be seen as an ongoing worldwide digitalization-process.

In both cohorts, “communication of medical findings”, “prescription” and “personal question” were the reasons mainly stressed for conducting a video consultation. Interestingly, there was a higher percentage for “personal question” seen in the Covid-19-cohort, which in our perspective can reflect a declining need for physical proximity to trust and open up towards the caring physician.

In the Covid-19 cohort, “availability from anywhere” as a reason in favor of the OVC, was significantly more often stated by the assessed patients than in the preCovid-19-cohort. The authors believe that this finding is underlining the decreasing need for personal contact to the doctor and the outside world during the corona crisis. Furthermore, “no danger of infection” was stated significantly more often as a potential advantage of the video consultation within the Covid-19-cohort. This finding is consistent with a previous report from Germany surveying 124 video consultation users, which found that the fear of a Covid-19-infection was the main reason for recently conducting a video consultation [25].

“Relationship between doctor and patient could change” and “no direct contact with the doctor” were significantly less stated as disadvantages of a potential video consultations amongst the patients in the Covid-19-cohort. In our believe, these findings again reflect a general fear of interpersonal interaction. Furthermore, it seems that general features of the physician-patient-interaction are of less importance since the coronavirus pandemic.

The present study has certain limitations. It is well known that surveys have minor level of evidence and the outcome of this study is directly connected to the participant’s understanding of the questionnaire. Furthermore, we did not assess the individual reasons for the patients’ visits to the respected outpatient clinics which could have biased their opinion on the feasibility of a potential video consultation. In addition, the chosen thresholds for the assessed age groups may not be optimal in view of age-related risk profiles of Covid-19.

We feel that our data support the rapid implementation of new remote tools in times of the Covid-19 pandemic and demonstrates the implications of the OVC s out of the patient’s point of view.

For both parties, patients and physicians, the pandemic has brought to light advantages and downsides of OVC. An ongoing stream of innovations in this field may find ways to cope with sometimes-anachronistic situations encountered during the attempts to establish a remote patient-physician communication in the year 2020. Future studies are necessary to assess and quantify the true value of OVC.

## Conclusion

More than half of the assessed orthopedic and trauma patients in this study would use an OVC. Covid-19 did not increase the disposition for an OVC significantly. However, a higher disposition towards the use of OVC was found amongst women since the Covid-19-pandemic. Fear of infections in the doctor’s office seems to have increased and a direct contact with the treating physician seems to be less important since the pandemic.

## Data Availability

Data are available upon reasonable request.
